# Heteroresistance to colistin in wild-type *Klebsiella pneumoniae* isolates from clinical origin

**DOI:** 10.1128/spectrum.02238-23

**Published:** 2023-11-14

**Authors:** Irene Sánchez-León, Elena Pérez-Nadales, Juan Antonio Marín-Sanz, Teresa García-Martínez, Luis Martínez-Martínez

**Affiliations:** 1 Maimonides Biomedical Research Institute of Cordoba, Cordoba, Spain; 2 Department of Agricultural Chemistry, Edaphology and Microbiology, University of Cordoba, Cordoba, Spain; 3 Centro de Investigación Biomédica en Red de Enfermedades Infecciosas (CIBERINFEC), Instituto de Salud Carlos III, Madrid, Spain; 4 Department of Computer Sciences, University of Cordoba, Cordoba, Spain; 5 Clinical Unit of Microbiology, Reina Sofía University Hospital, Cordoba, Spain; National Taiwan University, Taipei, Taiwan

**Keywords:** *Klebsiella pneumoniae*, heteroresistance, colistin, *mgr*B

## Abstract

Heteroresistance to colistin can be defined as the presence of resistant subpopulations in an isolate that is susceptible to colistin. The aim of this work was to study heteroresistance in 10 wild-type *Klebsiella pneumoniae* isolates of clinical origin, to determine the importance of persisters variants and stable mutants in heteroresistance, and to describe genomic changes observed in mutants obtained in population analysis profiling (PAP) assays. Mutations in genes related to colistin resistance (including *pmr*A, *pmr*B, *pmr*C, *pmr*D, *pho*P, *pho*Q, *crr*A, *ccr*B, *mgr*B, *ram*A, *rom*A, *lax*A, *lpx*C, *lpx*D, *acr*A, and *acr*B) were investigated by whole-genome sequencing with an Illumina platform. Additionally, PCR and Sanger sequencing of the *mgr*B gene of the selected mutants were also performed. PAP showed that the investigated *K. pneumoniae* isolates present heteroresistance to colistin, which is related to both the growth of persisters variants and the selection of resistant mutants. A total of 31-point mutations were identified in proteins PhoP, PhoQ, PmrB, and CrrB of which novel mutations were reported in PhoP (E22K, L12Q, and M175K), PhoQ (V24G and L105Q), PmrB (G207D), and CrrB (G183V). MgrB changes included amino acid substitutions (D31N and L19R), deletions (∆C28, ∆C39, ∆Q22, and ∆W6) or the presence of insertion sequences belonging to the IS1 (ISKpn14) and IS5 (ISKpn74) families that inactivated the gene. The results of this study will help to understand the mechanisms involved in colistin heteroresistance among wild-type *K. pneumoniae* isolates.

**IMPORTANCE:**

Colistin is one of the last remaining therapeutic options for dealing with Enterobacteriaceae. Unfortunately, heteroresistance to colistin is also rapidly increasing. We described the prevalence of colistin heteroresistance in a variety of wild-type strains of *Klebsiella pneumoniae* and the evolution of these strains with colistin heteroresistance to a resistant phenotype after colistin exposure and withdrawal. Resistant mutants were characterized at the molecular level, and numerous mutations in genes related to lipopolysaccharide formation were observed. In colistin-treated patients, the evolution of *K. pneumoniae* heteroresistance to resistance phenotype could lead to higher rates of therapeutic failure.

## INTRODUCTION

Polymyxin B and colistin (polymyxin E) were approved for clinical use in the late 1950s but fell out of favor during the mid-1970s owing to concerns over their potential to cause nephrotoxicity and neurotoxicity. More recently, polymyxins have been reconsidered as useful therapeutic agents for the treatment of infections caused by multiresistant gram-negative bacteria (1 – 3).

Colistin is a bactericidal agent interacting with the lipid A residue of lipopolysaccharide (LPS). After it penetrates into the bacterial cell, it causes disruption of the inner membrane, which leads to an exit of the cytoplasmic content and bacterial death (4). Resistance to colistin in *Klebsiella pneumoniae* is frequently related to LPS modifications caused by chromosomal mutations in genes coding for two-component systems (*pmr*A, *pmr*B, *pho*P, *pho*Q, *crr*A, and *crr*B) and their regulators (*mgr*B). These mutations activate bacterial genes by adding 4-amino-4-deoxy-Larabinose (*pmrHFIJKLM*) and/or phosphoethanolamine (*pmrC*) to lipid A of LPS. Colistin resistance has also been related to plasmid genes encoding MCR proteins (phosphoethanolamine transferases), the expression of active efflux pumps, the overexpression of capsular polysaccharides, and/or altered outer membrane proteins (5 – 12).

Heteroresistance can be defined as the presence of resistant subpopulations in an isolate that is susceptible to a given antimicrobial agent (13, 14). This has been related to the occurrence of persisters [organisms that can survive the lethal action of antibiotics without a change in their minimal inhibitory concentration (MIC)] (15, 16) or to the selection of stable mutants (stable increase in MIC) (17). Persisters can refer to an unstable subpopulation which can revert to the susceptibility of the original population in the absence of antibiotic, while in the stable subpopulation, mutations [such as single-nucleotide polymorphism (SNPs), insertions, or deletions] maintain increased MIC values (18). In *K. pneumoniae,* several studies have documented the presence of heteroresistant subpopulations, but they have more often evaluated multiresistant or carbapenemase-resistant clinical isolates (19 – 31).

The main aim of this study was to evaluate if clinical isolates of *K. pneumoniae* with a wild-type phenotype (resistant only to aminopenicillins; susceptible to colistin) express heteroresistance to colistin and if heteroresistance is related to either or both growth of persisters or selection of stable mutants. The presence of mutations in genes related to colistin resistance in stable mutants was studied by whole-genome sequencing (WGS).

## MATERIALS AND METHODS

### Bacterial strains

Ten wild-type clinical isolates of *K. pneumoniae* cultured from diagnostic samples of different patients admitted to the Reina Sofía University Hospital (Córdoba, Spain) were evaluated (Table 1). Bacterial identification was performed using a matrix-assisted laser desorption/ionization-time of flight mass spectrometry system (Bruker Biotyper). The isolates were stored at −80°C in tryptic soy broth with 10% glycerol until used in this study.

**TABLE 1:** Sequence types (ST) of 10 *K*. *pneumoniae* isolates with wild-type resistance phenotype and their corresponding MICs and MBCs of colistin

Isolate	Year and source of isolation	Wzi allele	ST	Colistin MIC (mg/L) by broth microdilution		Colistin MBC (mg/L)		Colistin MIC (mg/L) by gradient strip (Etest)	
				10^5^ CFU/mL	10^7^ CFU/mL	10^5^ CFU/mL	10^7^ CFU/mL	10^8^ CFU/mL	10^10^ CFU/mL
5–1534_P	2019, urethral isolate	95	ST268	0.25	16	1	16	0.125	0.25
9–1075_P	2019, organic fluid	349	ST17	0.06	16	0.125^*a*^	32	0.094	0.094
170652_P	2017, blood	236	ST34	0.06	16	0.125^*a*^	32	0.094	0.125
170943_P	2017, blood	236	ST3478	0.125	1	0.25	1^*a*^	0.094	0.125
171289_P	2017, blood	210	ST3477	0.125	8	0.5^*a*^	8	0.094	0.125
171503_P	2017, blood	85	ST29	0.06	1	0.06	1^*a*^	0.094	0.125
171703_P	2017, blood	419	ST1628	0.125	8	0.125^*a*^	8	0.125	0.25
174774_P	2017, blood	419	ST875	0.06	32	0.25^*a*^	64	0.125	0.125
174873_P	2017, blood	86	ST2436	0.25	8	0.25	16	0.094	0.125
175802_P	2017, blood	361	ST1825	0.125	2	0.125	2^*a*^	0.094	0.125

^*a*^ Eagle effect.

### Antimicrobial susceptibility testing

MICs of cefotaxime, imipenem, meropenem, ertapenem, levofloxacin, tigecycline (Discovery Fine Chemicals, Wimborne, United Kingdom), ceftazidime, cefepime, gentamicin, amikacin, tobramycin, netilmicin, nalidixic acid, and ciprofloxacin (Sigma-Aldrich, Steinheim, Germany) were determined by standardized broth microdilution (BMD) (32). MICs of colistin (Sigma-Aldrich) were also determined by microdilution, following the guidelines from the CLSI-EUCAST working group (33). MICs of colistin were determined using two different inocula (10^5^ and 10^7^ CFU/mL). Clinical categories were defined according to the European Committee of the Antimicrobial Suscetibility Testing (34). *Escherichia coli* ATCC 25922, *Pseudomonas aeruginosa* ATCC 27853, and *E. coli* NCTC 13846 (containing *mcr-1*) were used as control strains.

The activity of colistin was also tested with gradient strips (Etest, bioMérieux, Marcy l’Étoile, France) on Mueller-Hinton agar plates, using two different inocula (ca. 10^8^ and 10^10^ CFU/mL). Plates were incubated at 35°C ± 2°C up to 7 days to evaluate the possible emergence of colonies within the inhibition zones.

The minimum bactericidal concentration (MBC) for colistin was determined from the BMD plates, following CLSI guidelines (35). For this, 100 µL of all the wells of BMD plates without visible bacterial growth and the wells with the highest colistin concentration where growth was observed were subcultured on colistin-free Mueller-Hinton agar plates (one plate per well to avoid an antibiotic carryover effect). MBC was defined at 99.9% death of the initial inoculum.

### Determination of heteroresistance to colistin

The presence of bacterial subpopulations heteroresistant to colistin was determined by population analysis profiling (PAP) (4, 36). For this purpose, Mueller-Hinton agar plates containing colistin sulfate concentrations of 0.06, 0.125, 0.25, 0.5, 1, 2, 4, 8, 16, 32, and 64 mg/L were inoculated with 100 µL of a bacterial suspension prepared from an overnight tryptic soy broth culture (approximately 10^8^ CFU/mL). The plates were incubated at 35°C ± 2°C in air, and colonies were counted at 48 h, 5 days, and 7 days (however, during the assays, no relevant changes were observed on day 7 in comparison with day 5). Colistin heteroresistance was defined as the growth of a resistant subpopulation at antibiotic concentrations at least eightfold higher than the MIC of the parental isolate. Up to eight colonies from each plate containing colistin concentrations at 4 × MIC, 16 × MIC, and the highest concentration (MAX) where growth occurred were selected. Colonies were subcultured twice in colistin-free medium, and standardized BMD was performed again to detect persisters variants (their MIC was within one two-fold dilution of the MIC for the parental isolate) and/or stable mutants (at least a two twofold increase in their MIC). Finally, five mutants with MIC of colistin representative of the different values observed for the eight colonies per plate initially evaluated were selected for genomic studies (see below). The lower limit of quantification (LOQ) was 90 CFU/mL (i.e., 1.9 log10 CFU/mL) (18).

### Extraction of genomic DNA for WGS

Genomic DNA (gDNA) of the 10 wild-type parental isolates and the five resistant colonies derived from each wild-type parental isolate was extracted using the MagCore HF16 Plus automatic nucleic acid extractor and the MagCore Genomic DNA Bacterial Kit 502 extraction kit (RBCBioscience, New Taipei City, Taiwan), following manufacturer’s instructions. A final elution volume of 60 µL of purified gDNA was obtained. gDNA concentration was determined using the NanoDrop 2000/2000c spectrophotometer (Thermo Fisher Scientific, MA, USA).

### Sequencing and bioinformatic analysis

gDNA libraries and WGS were performed by Macrogen (Seoul, South Korea) using the Illumina MiSeq platform. Sequences of parental isolates were compared with the genome sequence of *K. pneumoniae* ATCC 13883. Sequences of the five mutants derived from each parental isolate were compared with the sequence of their parental isolate. Data were analyzed using an in-house pipeline. Quality analysis and trimming of reads were performed by FastQC (https://www.bioinformatics.babraham.ac.uk/projects/fastqc/) and TrimGalore (https://www.bioinformatics.babraham.ac.uk/projects/trim_galore/). Genome assembly was performed using UniCycler (https://journals.plos.org/ploscompbiol/article?id = 10.1371/journal.pcbi.1005595) and annotated by Rast (https://rast.nmpdr.org/). The search for genes of interest (*pmr*A, *pmr*B, *pmr*C, *pmr*D, *pho*P, *pho*Q, *mgr*B, *crr*A, *crr*B, *ram*A, *rom*A, *lax*A, *lpx*C, *lpx*D, *acr*A, and *acr*B) was performed by aligning reference sequences in the original isolates and then partitioning these original sequences into small overlapping seed fragments and using BLASTn search algorithm (https://blast.ncbi.nlm.nih.gov/Blast.cgi).

The PROVEAN tool (http://provean.jcvi.org, J. Craig Venter Institute, La Jolla, CA, USA) was used to predict the neutral or deleterious biological impact of amino acid substitutions and insertions/deletions on protein function (37). The ISfinder database (https://www-is.biotoul.fr) was used to identify insertion sequence elements. The isolates were typed by multilocus sequence typing MLST (38) v2.0 (Center for Genomic Epidemiology, Technical University of Denmark, Lingby, Denmark; https://cge.cbs.dtu.dk/services/MLST/) (39). *In silico* capsular typing via the *wzi* gene was performed using whole genome MLST databases (http://bigsdb.pasteur.fr/).

### Molecular characterization of *mgr*B

To characterize genetic alterations in *mgr*B genes, gDNAs were obtained from the isolates of interest and were subjected to PCR using specific primers spanning the promoter and open reading frame (ORF) of the gene (mgrB_U111_F: 5′ CGTTTTGAAACAAGTCGATGA 3′ and mgrB_D248_R: 5′ ATTCTGCCGCTTTTGCTG 3′), only the ORF (mgrB_013_F: 5′ CGGTGGGTTTTACTGATAGTCA 3′ and mgrB_0122_R: 5′′ATAGTGCAAATGCCGCTGA 3′), and primers covering the ORF and both the upstream and downstream regions (mgrB_U503_F: 5′ GCGCGTAAGATTTCTGAACAAATGGG 3′ and mgrB_D1229_R: 5′ ACCCTGGATAGCGGAGAAGT 3′) (40). The PCR product obtained was purified and sequenced (both strands) to identify mutations and insertions.

## RESULTS

### General susceptibility pattern

Among the 10 isolates of *K. pneumoniae*, 10 different sequence types (ST) were identified (Table 1). MICs of colistin determined by BMD were in the range of 0.06–0.25 mg/L using the reference inoculum of 10^5^ CFU/mL, but when a 10^7^ CFU/mL inoculum was used, 7 out of the 10 isolates presented MICs of colistin ranging 8–32 mg/L, and for the remaining three isolates, MICs also increased 8–16 times. MBC values were the same or up to one or two twofold dilutions higher than the corresponding MIC values for the reference inoculum (10^5^ CFU/mL); in 5 of the 10 isolates, an Eagle effect occurred. For a 10^7^ CFU/mL inoculum, MBCs were the same (six isolates) or one twofold dilution higher (four isolates) than the corresponding MICs, and, again, in three isolates the Eagle effect appeared. When Etest strips were used with an inoculum of 10^8^ CFU/mL, the MICs ranged from 0.094 to 0.125 mg/L. Similar results were observed (within a one twofold dilution) with an inoculum of 10^10^ CFU/mL. Colonies did not grow up within the inhibition zones of the gradient strips inoculated with either inocula for any of the isolates.

### Analysis of colistin heteroresistance in wild-type *K. pneumoniae*: PAP assays

**Fig 1:**
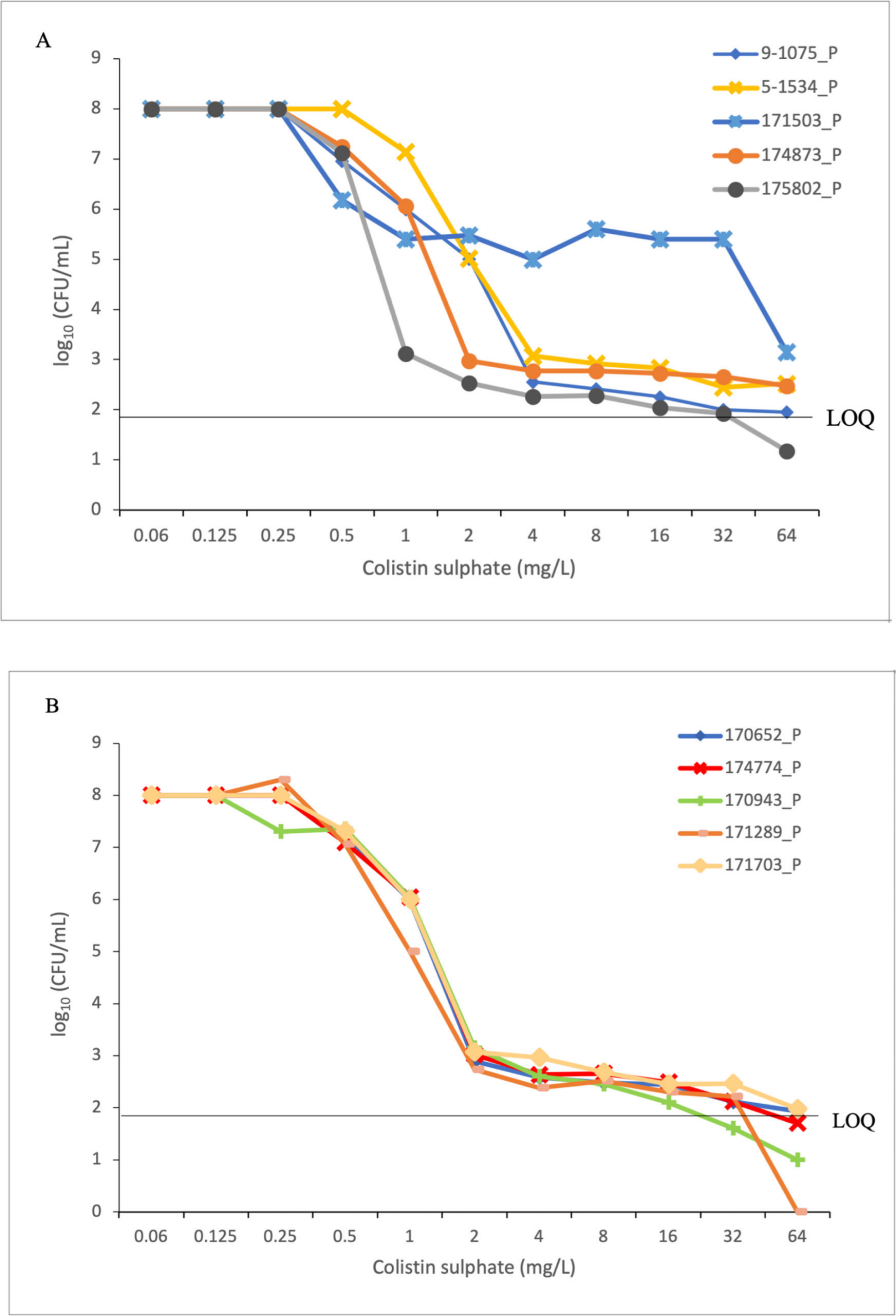
(A and B) PAP of 10 selected wild-type isolates. The graphs represent the log10 CFU/mL on the different colistin concentrations relative to the plated viable counts. The lower LOQ was 90 CFU/mL (i.e., 1.9 log10 CFU/mL).

Unlike the results observed with gradient strips, all 10 parental *K. pneumoniae* isolates herein evaluated showed heteroresistance to colistin when assayed with the PAP method. In all cases, colonies grew on plates with high colistin concentrations [up to 32 mg/L (isolate 171289_P) or 64 mg/L (the remaining nine isolates); (Fig. 1A and B)]. The PAP curves of the parental isolates did not show different trends among them, except for the curve of the parental isolate 171503_P which showed a growth of 10^5^ CFU/mL in concentrations of 1–32 mg/L (Fig. 1A). In all isolates, at concentrations of 0.25–4 mg/L colistin, there was a decrease in growth of approximately 10^5^ CFU/mL, but they continued to grow at concentrations of 2 to 32 mg/L, with colony counts in the range of 10^2^–10^3^ CFU/mL.

For all parental isolates, the colistin MICs for isolates grown on PAP plates with colistin concentrations of 16 × MIC or MAX were 16–128 mg/L (Table 2), indicating the presence of stable mutant subpopulations. On the other hand, in 6/10 parental isolates, MICs for two to four colonies grown in PAP plates with 4 × MICs of colistin (absolute values ranging 0.06 to 0.25 mg/L) were similar to the original MICs (colistin-susceptible) and, therefore, were defined as persisters variants.

**TABLE 2:** MIC of colistin for the colonies of *K. pneumoniae* isolates selected on PAP plates containing the indicated colistin concentration

		5–1534_P	9–1075_P	170652_P	174774_P	170943_P	171289_P	171503_P	171703_P	174873_P	175802_P
	MIC (mg/L)	0.25	0.25	0.125	0.06	0.06	0.125	0.06	0.125	0.125	0.125
Colistin concentrations	Mutant	MIC (mg/L)	MIC (mg/L)	MIC (mg/L)	MIC (mg/L)	MIC (mg/L)	MIC (mg/L)	MIC (mg/L)	MIC (mg/L)	MIC (mg/L)	MIC (mg/L)
4 × MIC	1	4	64	128	8	≤0.06^*b*^	0.125^*b*^	32	16	32	8
	2	1	32	>128	16	16	0.125^*b*^	64	2	0.125^*b*^	16
	3	8	8	16	32	≤0.06^*b*^	32	64	16	16	64
	4	4	0.25^*b*^	>128	8	8	1	64	8	0.125^*b*^	32
	5	0.5^*b*^	0.25^*b*^	>128	1	0.125^*b*^	32	64	16	32	32
	6	0.5^*b*^	1	16	0.125^*b*^	0.125^*b*^	32	64	16	0.25^*b*^	16
	7	4	32	16	16	16	≤0.06^*b*^	16	16	0.125^*b*^	16
	8	16	64	32	0.125^*b*^	32	≤0.06^*b*^	64	16	16	16
16 × MIC	1	64	32	32	16	16	64	16	16	16	16
	2	1	64	>128	16	8	8	16	32	16	16
	3	16	64	>128	16	8	16	128	16	16	16
	4	1	32	128	8	16	32	32	>128	16	16
	5	16	64	>128	32	8	16	64	32	16	32
	6	1	64	>128	8	16	32	16	64	16	16
	7	64	64	>128	16	8	32	32	8	16	32
	8	8	64	64	32	16	32	32	32	32	32
[MAX]^*a*^	1	64	64	16	>128	32	32	16	32	32	32
	2	32	64	128	128	128	32	64	32	16	32
	3	64	64	>128	32	−^*c*^	32	64	32	16	16
	4	32	>128	16	16	−	32	64	32	32	−
	5	64	64	16	>128	−	32	32	32	16	−
	6	64	64	128	32	−	32	64	16	16	−
	7	64	>128	128	32	−	16	64	16	32	−
	8	32	64	16	16	−	16	64	32	16	−

^*a*^ [MAX], the highest concentration where growth was selected.

^*b*^ Persister variants.

^*c*^ −, no colony appeared.

### Genetic analysis of genes involved in colistin resistance

Fifty stable mutants selected in the PAP assays were studied to identify the putative mechanisms of acquired resistance to colistin. Fourteen (28%) of them did not show any genetic modifications (when compared with the corresponding parental susceptible isolates) in the genes related to colistin resistance evaluated in this study.

**Fig 2:**
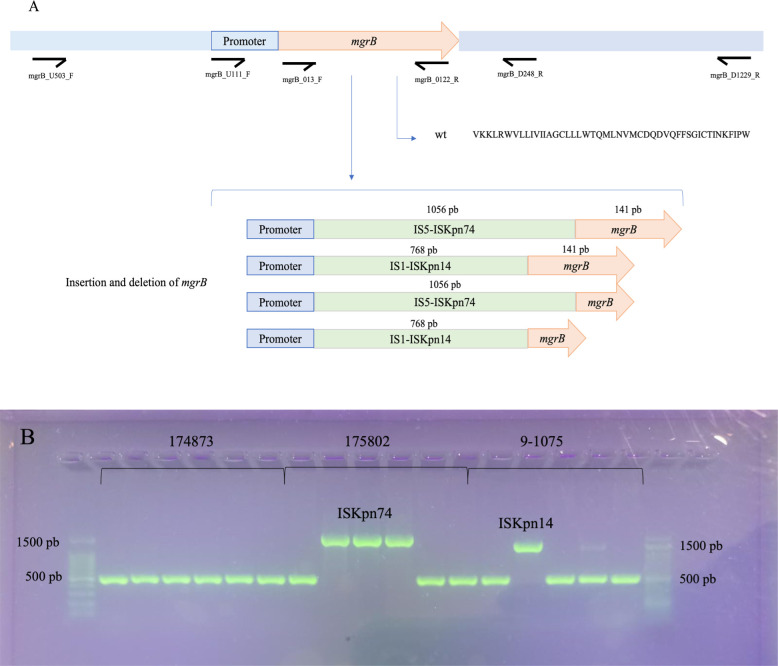
(**A**) *mgrB* alterations mediated by insertion of IS elements and mutations. (**B**) Example of agarose gels of three wild-type isolates and their mutants.

MgrB is a small transmembrane protein that inhibits PhoQ kinase activity. Mutations in this protein result in the overexpression of the *pho*PQ operon leading to an excess of the cationic component in lipid A. The MgrB protein showed amino acids alterations in 54% (27/50) resistant mutants (Table 3). In 11 of these isolates, the genetic changes resulted in amino acid substitutions (D31N and L19R) or deletions (∆W6, ∆Q22, ∆C28, and ∆C39) in the MgrB protein. All these mutations were defined as deleterious by PROVEAN analysis (Table 4). In 14 (28%) mutants, amplification of the *mgr*B gene with primers mgrB_U111_F and mgrB_D248_R (flaking the ORF) identified two bands of approximately 1,200 pb and 1,500 bp that matched an insertion sequence that inactivated the gene. These insertions were identified as belonging to the IS1 (ISKpn14; 8/50, 16%) and IS5 (ISKpn74; 6/50, 12%) families and were inserted as a single fragment into the ORF resulting in deletion in part of the gene or between the promoter and the ORF (Table 3; Fig. 2). Using the different primers described above, there was no amplification of the *mgrB* gene in two isolates (4%), which was probably related to the loss of this region during a deletion event. The absence of the *mgrB* gene of these two mutants was confirmed in the annotated genome. CrrA–CrrB were not detected in four parental isolates. The *crr*AB (colistin resistance regulation) operon codes for two proteins: the regulatory protein CrrA and the sensor protein kinase CrrB. *crr*AB acts as a positive regulator by activating *pmr*C gene expression through PmrAB. No changes in any mutant were observed in proteins PmrA, PmrC, PmrD, or CrrA.

**TABLE 3:** Phenotypic and genotypic characteristics of the 10 parental and 50 mutants studied *K. pneumoniae* isolates

	Isolates	MIC (mg/L)	*mgr*B	MgrB	PhoP	PhoQ	PmrB	CrrA	CrrB
Isolates	ATCC 13883	1	Ref	Ref	Ref	Ref	Ref	Ref	Ref
	170652_P	0.06	−^*b*^	−	−	−	−	−	P73L
	170943_P	0.13	−	−	−	−	−	−	P73L
	171289_P	0.13	−	−	−	R64k, Q92R, V196I, G465S, and Q482L	M175v, N244S, V247I, Q356R, and G358A	NF^*a*^	NF
	171503_P	0.06	−	−	−	−	−	−	I66v, Q239H, T276A, and Q287K
	171703_P	0.13	−	−	−	−	R256G	−	I66v and K325R
	174774_P	0.06	−	−	−	−	R256G	−	I66v and K325R
	174873_P	0.25	−	−	−	−	A246T	NF	NF
	175802_P	0.13	−	−	−	H234R	−	NF	NF
	5–1534_P	0.25	−	−	−	−	−	NF	NF
	9–1075_P	0.06	−	−	−	−	−	−	−
170652_P	170652_D4_MICH128	>128	Codon 28 (TGC>TGA stop)	∆C28	−	−	−	−	−
	170652_D3_MIC128	128	−	−	−	−	−	NF	−
	170652_D5_MICH128	>128	−	−	−	−	−	−	−
	170652_D2_MIC64	64	IS5_ISKpn74	V1_V7del	−	V24G	−	−	−
	170652_D1_MIC32	32	−	−	−	−	−	−	−
170943_P	170943_D4_MIC32	32	IS5_ISKpn74	−	−	−	−	−	−
	170943_D2_MIC16	16	−	−	E22K	−	−	−	−
	170943_D5_MIC128	128	−	−	L12Q	−	−	−	−
	170943_D1_MIC8	8	Codon 31 (GAT>AAT)	D31N	−	−	−	−	−
	170943_D3_MIC16	16	−	−	−	−	−	−	−
171289_P	171289_D5_MIC32	32	−	−	−	−	−	NF	NF
	171289_D2_MIC8	8	−	−	−	−	−	NF	NF
	171289_D3_MIC16	16	−	−	−	−	−	NF	NF
	171289_D4_MIC32	32	IS1_ISKpn14	−	−	−	−	NF	NF
	171289_D1_MIC1	1	−	−	−	−	−	NF	NF
171503_P	171503_D5_MIC128	128	No amplicon	−	−	−	−	−	−
	171503_D4_MIC64	64	No amplicon	−	−	−	−	−	−
	171503_D2_MIC32	32	IS1_ISKpn14	−	−	−	−	−	−
	171503_D1_MIC16	16	IS1_ISKpn14	−	−	−	−	−	−
	171503_D3_MIC64	64	IS1_ISKpn14	−	−	−	−	−	−
171703_P	171703_D3_MIC32	32	Codon 39 (TGC>TGA stop)	∆C39	−	−	−	−	−
	171703_D1_MIC8	8	−	−	−	S43_F44in QAVSHFL THWLDNPA	−	−	G183V
	171703_D5_MICH128	>128	−	−	−	−	−	−	−
	171703_D2_MIC16	16	−	−	M175K	−	−	−	−
	171703_D4_MIC64	64	Codon 22 (CAG>TAG stop)	∆Q22	−	−	−	−	−
174774_P	174774_D1_MIC8	8	−	−	−	−	G207D	−	−
	174774_D5_MICH128	>128	Codon 19 (CTG>CGG)	L19R	−	−	−	−	−
	174774_D2_MIC16	16	Codon 19 (CTG>CGG)	L19R	−	−	−	−	−
	174774_D4_MIC128	128	IS1_ISKpn14	−	−	−	−	−	−
	174774_D3_MIC32	32	IS5_ISKpn74	V1_I12 del	−	−	−	−	−
174873_P	174873_D2_MIC16	16	∆nt8 (A)	∆W6	−	−	−	NF	NF
	174873_D4_MIC32	32	Codon 6 (TGG>TGA stop)	∆W6	−	−	−	NF	NF
	174873_D5_MIC32	32	∆nt8 (A)	∆W6	−	−	−	NF	NF
	174873_D3_MIC16	16	Codon 6 (TGG>TGA stop)	∆W6	−	−	−	NF	NF
	174873_D1_MIC16	16	∆nt8 (A)	∆W6	−	−	−	NF	NF
175802_P	175802_D1_MIC8	8	−	−	−	L105Q	−	NF	NF
	175802_D4_MIC32	32	IS5_ISKpn74	V1_W20 del	−	−	−	NF	NF
	175802_D5_MIC64	64	−	−	L12Q	−	−	NF	NF
	175802_D2_MIC16	16	IS5_ISKpn74	V1_I12 del	−	−	−	NF	NF
	175802_D3_MIC32	32	IS5_ISKpn74	V1_T21 del	−	−	−	NF	NF
5–1534_P	5–1534_D1_MIC4	4	−	−	−	−	−	NF	NF
	5–1534_D5_MIC64	64	IS1_ISKpn14	−	−	−	−	NF	NF
	5–1534_D4_MIC32	32	IS1_ISKpn14	T40_W47del	−	−	−	NF	NF
	5–1534_D2_MIC8	8	−	−	−	−	−	NF	NF
	5–1534_D3_MIC16	16	−	−	−	−	−	NF	NF
9–1075_P	9–1075_D3_MIC32	32	−	−	−	−	T157P	−	−
	9–1075_D2_MIC8	8	−	−	−	−	−	−	−
	9–1075_D4_MIC64	64	IS1_ISKpn14	T40_W47del	−	−	−	−	−
	9–1075_D5_MICH128	>128	−	−	−	K46E	−	−	−
	9–1075_D1_MIC1	1	−	−	−	−	−	−	−

^*a*^ NF, not found.

^*b*^ −, mutations not detected.

**TABLE 4:** Analysis of mutations in colistin-resistance related genes using PROVEAN tool

Protein	Variant	PROVEAN score	Prediction (cutoff = 2.5)
PhoQ	V24G	4.762	Deleterious
	K46E	2.110	Neutral
	R64K	0.274	Neutral
	Q92R	0.472	Neutral
	L105Q	2.264	Neutral
	V196I	0.066	Neutral
	H234R	0.452	Neutral
	G465S	0.363	Neutral
	Q482L	1.826	Neutral
	S43_F44insQAVSHFLTHWLDNPA	17.226	Deleterious
PhoP	L12Q	5.685	Deleterious
	E22K	1.295	Neutral
	M175K	1.968	Neutral
MgrB	L19R	5.908	Deleterious
	D31N	4.143	Deleterious
	∆W6	18.683	Deleterious
	∆Q22	14.615	Deleterious
	∆C28	19.000	Deleterious
	∆C39	18.857	Deleterious
	V1_V7del	24.101	Deleterious
	V1_I12del	37.940	Deleterious
	T40_W47del	41.399	Deleterious
PmrB	T157P	5.787	Deleterious
	M175V	0.784	Neutral
	G207D	5.793	Deleterious
	N244S	0.871	Neutral
	A246T	1.132	Neutral
	V247I	0.509	Neutral
	R256G	5.484	Deleterious
	Q356R	0.290	Neutral
	G358A	1.022	Neutral
CrrB	G183V	8.575	Deleterious

The *pho*PQ and *pmr*AB systems are upregulated in *K. pneumoniae* exposed to polymyxins indicating that these systems are involved in polymyxin resistance in this bacterium. The constitutive activation of the *pho*PQ or *pmr*AB system can also be caused by missense mutations, leading to the subsequent upregulation of *pmr*C and the *arn*BCADTEF operon, resulting in the synthesis and addition of PEtN and L-Ara4N, respectively, to lipid A (cationic component). PmrB is the transmembrane sensor kinase that activates PmrA which, in turn, activates other lipid A modification genes. For the PmrB protein, amino acid changes were shown in two positions (G207D and T157P) of two (4%) mutants. Both changes were predicted to be deleterious by the PROVEAN tool.

Modifications in the proteins of the double component system PhoP and PhoQ were found in 16% (8/50) of the resistant mutants. For the PhoP protein, three amino acid substitutions (E22K, L12Q, and M175K) were identified in three mutants selected from three different parental isolates (170943_P, 171703_P, and 175802_P, respectively); only the L12Q mutation was defined as deleterious according to PROVEAN analysis. PhoQ also showed three amino acid substitutions (V24G, L105Q, and K46E, of which only V24G was predicted as deleterious by PROVEAN analysis). Finally, we identified an IS3 insertion in mutant 171703_D1_MIC8 that completely inactivated the protein (Table 3). This isolate also showed a deleterious (PROVEAN) amino acid change (G183V) in the CrrB protein.

To investigate the diversity of mutations in additional putative genes responsible for increasing colistin MICs in the laboratory-evolved mutants, pair-wise genetic relatedness among mutants and their corresponding parental wild type was analyzed using whole-genome SNP data. A total of around 200 genes carrying non-synonymous SNPs were identified among the 50 mutants. Three hundred and thirty-three nron-synonymous SNPs were detected in mutants (10 in mutants derived from parental 170652_P, 17 from 170943_P, 26 from 171289_P, 20 from 171503_P, 67 from 171703_P, 47 from 174774_P, 63 from 174873_P, 11 from 175892_P, 55 from 5 to 1,534_P, and 17 from 9 to 1,075_P). Prediction of putative gene function was identified using Kyoto Encyclopedia of Genes and Genomes classification. The most common were genes involved in two-component systems (*n* = 3), metabolism-associated genes (*n* = 43), membrane transporters (*n* = 22), and DNA replication and repair genes (*n* = 121).

## DISCUSSION

In this study, several pharmacodynamic effects related to colistin interactions with wild-type *K. pneumoniae* have been observed. When microdilution was performed with a 10^7^ CFU/mL inoculum, MICs of colistin were much higher (8–512 times) than when using the standard 10^5^ CFU/mL inoculum. This inoculum effect has already been described for several pathogens, including *K. pneumoniae* (41 – 44). In *E. coli*, resistance to colistin related to an inoculum effect has a negative therapeutic impact, as demonstrated in a peritonitis model in mice (45). Although gradient strips are not considered reliable for conventional *in vitro* testing of colistin, in this study, and for the relatively small number of isolates evaluated, MICs of colistin determined by gradient strips were similar (0.06–0.25 mg/L) to those obtained with the reference microdilution assay and the conventional 10^5^ CFU/mL inoculum. Interestingly, and in contrast to the observation with BMD, MICs of colistin did not change when using gradient strips with a high inoculum, although the causes of this observation are undefined. It would be possible that it is related to an adaptive resistance occurring in broth dilution (42) but not on solid media. MBCs values demonstrate the expected bactericidal activity of colistin, but for some strains, an Eagle effect was noted. The Eagle effect has been previously documented for polymyxins and *Acinetobacter baumannii* (46, 47), but its mechanistic and genetic causes remain largely unknown. The Eagle effect resembles to bacterial persistence, in which a small population resists the bactericidal action (less than 0.1%), and it could be due to the action of autolysins that hydrolyze cell wall components contributing to bacterial death by antibiotics (46). Additional studies should explore the possibility that the inoculum and Eagle effects of colistin observed in this study with BMD might be related to the heteroresistance defined by PAP.

Detection of heteroresistance in the clinical laboratory is challenging. PAP is a widely accepted methodology for this purpose, but it is difficult to implement in daily work because of its technical complexity. The diffusion assay using gradient strips has been used in multiple studies aimed to detect heteroresistance to colistin because of its easy implementation, but only in a minority of cases it has been able to detect colistin-heteroresistant *K. pneumoniae* isolates. In this study, gradient strips were unable to detect colistin heteroresistance, as no colonies were detected within the inhibition zones of PAP-proven heteroresistant isolates, even a high-density inoculum was used and plates were incubated for as long as 7 days.

Most reports on heteroresistance to colistin in *K. pneumoniae* have been related to strains producing carbapenemases or extended-spectrum beta-lactamases (26, 28, 48 – 51), and only occasionally, heteroresistance has been described in non-multiresistant isolates. In this study, using PAP, we have shown that all 10 wild-type isolates of *K. pneumoniae* obtained from patients are heteroresistant to colistin. In most cases, heteroresistance in the wild-type *K. pneumoniae* clinical isolates herein evaluated was related to selection of stable mutants, but in some isolates, persisters were also able to grow at colistin concentrations as low as 0.06–1 mg/L (below the breakpoint for susceptibility). The emergence of persisters can be related to unstable tandem repeats, but this has not been observed in this study. It can be speculated that persisters growing at those low concentrations can represent a subpopulation favoring the selection of stable mutants. Additional studies are warranted to explore this hypothesis.

The analysis of colonies growing on plates with colistin confirmed that most of them are stable mutants. Strains of *Acinetobacter*, *Klebsiella*, or *S. typhimurium* have been identified showing stable heteroresistance that is maintained in the absence of antibiotic pressure (6, 18, 52 – 54). In this study, point mutations, deletions, and insertions were identified in multiple proteins related to colistin resistance (MgrB, PhoP, PhoQ, PmrB, and CrrB). Plasmid-encoded *mcr* genes were not detected in any isolates, demonstrating that resistance was related to chromosomally encoded mechanisms. Mutations in MgrB, a small transmembrane protein that inhibits PhoQ kinase activity, result in the overexpression of the *pho*PQ operon leading to an excess of the cationic component in lipid A. Point substitutions (D31N and L19R), deletions (∆C28, ∆C39, ∆Q22, and ∆W6), and IS elements were identified producing a non-functional protein. Some of these mutations have already been reported (40, 55 – 58), but to our knowledge, others (L19R) have been identified for the first time in our mutants. IS1-like (768 bp) and IS5-like (1,056 bp) elements inserted in different positions of the *mgr*B gene caused a disruption of this gene. In 1/50 mutant, IS5 was inserted between the start codon and the promoter region, as already reported (40, 55, 57, 58), but in 5/50 mutants, the IS5 element (ISKpn74) was inserted into the coding region of the *mgr*B gene, producing a simultaneous deletion of the start of the gene (from 36 to 63 pb), an event which has not been previously described. In another 2/50 resistant mutants, ISKpn14 (IS1 family) was inserted in the *mgr*B coding region with a deletion of 24 nucleotides, similar to the event described by Wright et al. (59) of an IS1 insertion and a 14 bp deletion in *mgr*B. Moreover, in 5/50 isolates, the IS1 insertion occurred between the start codon and the promoter region of the *mgr*B gene. Finally, in 2/50 resistant mutants, the absence of a *mgr*B gene amplicon indicated a complete gene deletion.

Although several point mutations in PhoP (E22K, L12Q, and M175K) have been identified in our study, only L12Q has been predicted as deleterious. Other authors have also identified other mutations (E96K and R48K) in colistin-resistant *K. pneumoniae* (60). Previous studies have described different mutations in PhoQ, including D152N, R249G, or F373L (defined as neutral not affecting the function of the protein) or K46E, L322V, or G385C (defined as deleterious producing a non-functional protein). We have also observed novel mutations (V24G y L105Q) and an insertion (IS3) producing a non-functional protein PhoQ.

Some of the investigated mutants presented predicted deleterious mutations in PmrB (G207D and T157P) or CrrB (G183V). In fact, the T157P change in PmrB has already been proven to be a cause of colistin resistance (60, 61). Deletions and different mutations in the CrrB protein were also described in previous studies (60, 62, 63).

This study indicates that colistin heteroresistance is a usual trait in *K. pneumoniae*, with independence of resistance derived from acquired mechanisms. Although, from a therapeutic perspective, colistin heteroresistance might not be critical when considering wild-type isolates (infections caused by these organisms can be treated with many other agents), it is a biological trait of potential clinical consequences when wild-type isolates acquire novel resistance traits compromising usual therapeutic alternatives.

## Data Availability

The data presented in this study have been deposited in the European Nucleotide Archive (ENA) repository under project number PRJEB66360.
